# Effect of Acute Aerobic Exercise on Inhibitory Control of College Students with Smartphone Addiction

**DOI:** 10.1155/2021/5530126

**Published:** 2021-08-05

**Authors:** Hainan Fan, Shuai Qi, Guoyuang Huang, Zhao Xu

**Affiliations:** ^1^School of Sport and Health, Shandong Sport University, Jinan, China; ^2^Pott College of Science, Engineering and Education, University of Southern Indiana, Evansville, IN, USA

## Abstract

**Background:**

Inhibitory control deficits may be one important cause for smartphone addiction. The available studies have shown that acute aerobic exercise may improve the inhibitory control. However, there is still lack of research on how regimens of an acute exercise affect this inhibitory control. The present study was to examine the effects of an acute aerobic exercise at three different exercise intensities on changes in the inhibitory control function including response inhibition and interference control in college students with smartphone addiction.

**Methods:**

Participants (*n* = 30; age 20.03 ± 0.96 years) with smartphone addiction were identified by the Mobile Phone Addiction Tendency Scale for College Students and randomized to study 1 and study 2 with 15 individuals each. Fifteen participants in study 1 were tested by the Go/NoGo task to explore the response inhibition, while other fifteen in study 2 were tested by the Flanker task to examine the interference control. The participants in study 1 and 2 were randomly assigned to three groups (5 in each) with exercising at low, moderate, and high intensity. The individual response inhibition and interference control were measured before and after 30 minutes acute aerobic exercise, respectively.

**Results:**

In study 1, the accuracy of NoGo stimulus after 30 minutes of acute aerobic exercise was significantly increased (*p* ≤ 0.001) while the response time (RT) of Go stimulus was significantly decreased (*p* ≤ 0.001). The largest changes occurred in the moderate-intensity group for the accuracy of NoGo stimulus (*p*=0.012) and for the RT of Go stimulus (*p* ≤ 0.001). The results in study 2 showed no significant change in all three groups after exercise.

**Conclusions:**

30 minutes of acute aerobic exercise could effectively elicit changes of the response inhibition in college students with smartphone addiction. The largest improvement was observed in the moderate intensity of an acute aerobic exercise in college students with smartphone addiction.

## 1. Introduction

As smartphones nowadays provide a high-degree of connectivity and open the doors to a new world for their users, addiction to smartphone usage is becoming a common problem among users worldwide. This form of addiction to information technology tools is characterized by excessive or compulsive mobile phone use and a preoccupation with the usage day by day or even all the day. Smartphone addiction could result in individuals losing control over their usage habits and/or turning smartphone use into a harmful activity on daily life. Consequently, this smartphone addiction interferes with or impairs an individual's daily functioning and brings negative physical and mental consequences [1–3]. For example, studies showed that the higher the degree of addiction to mobile phones, the more likely it would show problems such as the loss of normal proprioception and the unhealthy percentage of muscle and fat mass [4, 5]. This is the case, especially, in the young population. The young individuals are thought to be under risk of smartphone addiction because they have been living most of their lives in an environment where information technology has become so popular and ubiquitous [6]. For college students, it was reported that smartphone addiction could seriously affect their emotion, personality, interpersonal communications, and learning achievements [7–10]. A study from Cao reported that 51.81% of college students had significantly reduced their academic performance due to excessive use of smartphones [11].

Research has indicated the influence of smartphone addiction on psychological health. Similar to other behavioural addictions, losing control of psychological behaviour due to smartphone addiction may induce loneliness, depression, impulse, and low self-control [4, 6, 8, 12–14]. Some recent studies reported that college students with smartphone addiction could have significant inhibitory control deficits [15–17]. Inhibitory control involves in being able to control one's thoughts, behavior, and/or emotions to override a strong internal tendency or external lure and select to do what is more appropriate or needed [18]. Thus, it is important for researchers to explore the ways to improve the inhibitory control function for college students with mobile phone addiction.

Recently, one research attention in exercise psychology has gone to the effect of acute aerobic exercise on cognition function. An acute aerobic exercise refers to a single bout of exercise that can stimulate the body doing functional responses and changes. Current studies showed that acute aerobic exercise could improve the inhibitory control in healthy children, adolescents, and adults [19–24]. However, even for these populations, there are still disputes about the effect of exercise regime on the inhibitory control, for example, which intensity of an acute aerobic exercise would be more effective and significantly improve the function. Some studies reported that all intensities of acute aerobic exercise could significantly improve the inhibitory control [25, 26]. Other investigations, however, showed that the acute aerobic exercise at moderate intensity had the most obvious effect on inhibitory control, but the exercise at low or high intensity had a lower or little effect, showing an inverted-U-shaped curve relationship [27–29]. Interestingly, Schmit [30] reported that the inhibitory control would be affected by different intensities only in the early-stage exercise, while the high intensity at the end of the acute aerobic exercise could decrease the inhibitory control. Unfortunately, there is still lack of research on the effect of acute aerobic exercise on the inhibitory control and how regimens of an acute exercise affect this function among college students with smartphone addiction.

Inhibitory control function has been recognized including two components, response inhibition and interference control [18, 31]. They can be measured by different tasks [32]. The response inhibition refers to resisting temptations and resisting impulsive actions that are usually measured by Go/NoGo or Stop-Signal tasks [18]. The interference control means fostering resistance to interference from extraneous stimuli, which is usually measured by the Flanker task or Simon task [18]. As such, different natures of cognitive tasks could be used to provide a possible answer to the abovementioned disputes and to identify different effects of the acute aerobic exercise at different intensities on inhibitory control. For example, the Go/NoGo task was used in the study of Wang and Zhou [29], while Sandoff applied the Flanker task in their study [26]. Based on these considerations, the present study was to use both of the Go/NoGo task and Flanker task in two separate experiments to evaluate the effect of an acute aerobic exercise at different intensities on inhibitory control in college students with smartphone addiction. Our aims were to examine whether an acute aerobic exercise could improve the response inhibition and interference control in college students with smartphone addiction and to clarify if it did so, which intensity would be more effective.

## 2. Materials and Methods

**Figure 1:**
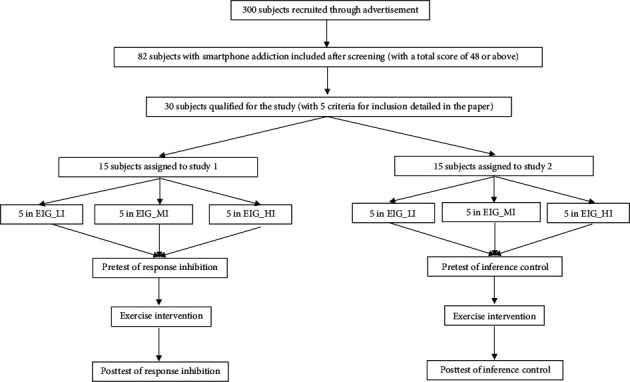
Flowchart of the study. EIG, exercise intervention groups; LI, low intensity; MI, moderate intensity; HI, high intensity.

The flowchart of study is shown in Figure 1. The study followed the guidelines which are in compliance with institutional, national, or international guidelines. The study protocol was reviewed and approved by the Ethical Committee of Shandong Sport University (No. 2019042).

### 2.1. Subject Recruitment

Through advertisement, 300 college students from two universities were initially recruited. All students underwent a screening survey, by using the Mobile Phone Addiction Tendency Scale for College Students (MPATS), to be identified as those with smartphone addiction [33]. The MPATS has 16 items scoring with Likert 5 points, total of 80 points. Those with a total score below 47 were normal mobile phone users, while those with a total score of 48 or above were considered to smartphone addiction [33]. A score indicated a higher degree of the smartphone addiction. The internal consistency coefficient of the scale was 0.829.

From 300 questionnaires, 296 returned as valid questionnaires with four invalid (three incomplete and one incorrect). From 296 students, 82 were identified with smartphone addiction as potential participants, accounting for 27.70%. Criteria for the research inclusion were (1) age ≥ 18 years; (2) no participation in any similar research project before; (3) normal vision or corrected vision and no colour blindness or colour weakness; (4) without cardiovascular, metabolic, renal, or pulmonary diseases or symptoms or psychological disorders; and (5) no regular aerobic exercise (less than 60 minutes per week) in the past six months. Thus, 30 out of 82 students were qualified for this study.

Prior to the testing, all subjects were asked to attend an orientation in the laboratory. They were instructed to familiarize with the experimental procedures and measurements to be applied in the study. After the investigators addressed the study purpose, method, and requirements, all participants agreed to voluntarily participate in the study and gave the informed consents. They were randomly divided into six groups by using Excel soft, with 5 participants in each group as follows: the randomly ordered number 1–5 to Study 1 Low Intensity (S1LI), 6–10 to Study 1 Moderate Intensity (S1MI), 11–15 to Study 1 High Intensity (S1HI), 16–20 to Study 2 Low Intensity (S2LI), 21–25 to Study 2 Moderate Intensity (S2MI), and 26–30 to Study 2 High Intensity (S2HI), respectively. Three groups were assigned into study 1, and the other three were assigned into study 2. All groups in study 1 and 2 conducted exercise intervention at three different levels of intensity. Participants in S1LI and S2LI were required to exercise reaching to 35～50% of their maximum heart rate (HRmax). Individuals in S1MI and S2MI were required to reach 50～70% of their HRmax. Subjects in S1HI and S2HI should exercise reaching to 70～85% of their HRmax. The used formula for the estimated HRmax was 207–0.7 × age [34].

### 2.2. Study 1

A total of 15 college students with smartphone addiction voluntarily participated in study 1 (males, *n* = 9; females, *n* = 6) with an average age of 20.20 ± 0.94 years. Written informed consent was obtained from each subject.

#### 2.2.1. Pretest of Response Inhibition

**Figure 2:**
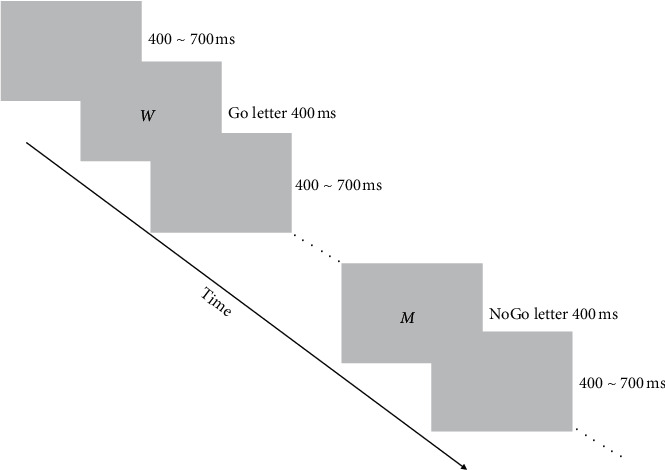
Process of response inhibition testing for study 1.

The experiment was carried out on a computer with an Intel central processor. The display was Qimei 8201, the screen resolution was 1920 × 1200, and the refresh frequency was 60 Hz. The experiment task was programmed and run on E-Prime software. The distance between the eyes and the center of the screen was 57 cm. The Go/NoGo task [29] was used to measure the response inhibition of college students with smartphone addiction. The measured variables were reaction time (RT) and accuracy. The process is shown in Figure 2. At the beginning of testing, a series of stimulus characters was presented in the center of the screen one by one. The presentation time of each stimulus character was 400 ms, and the interval time between the stimulus characters was random, 400～700 ms. Before testing, the participants were told that “*M*” was a Go stimulus and “*W*” was a NoGo stimulus. When “*M*” appeared in the center of the screen, the participants were required to press the “*J*” key with their index finger as soon as possible under the premise of accuracy, while when “*W*” appeared, the participants were required to do nothing. The response time and accuracy of Go stimulation and the accuracy of NoGo stimulation were recorded. The total number of stimulus characters was 240, with 180 times of Go stimulus (75%) and 60 times of NoGo stimulus (25%) [29]. Each participant had opportunities to practice, but the formal experiment could be allowed to carry out only when the accuracy was over 90%.

#### 2.2.2. Exercise Intervention

Before exercise intervention, the resting heart rate of each participant was measured. The RS400 heart rate telemeter of Finland Polar was used to monitor the heart rate of participants in real time of testing. Participants performed an acute aerobic exercise using a bicycle ergometer (Ergoline, Germany). The acute aerobic cycling exercise lasted for 30 minutes [29]. The exercise loads were set manually. The bicycle power was set to 50 W with revolution speed at 50 rpm and exercise time for 30 minutes. According to the specific physical conditions of each participant, the resistance was adjusted to keep each individual's heart rate within his/her corresponding heart rate range. Exercise intervention began with a 5-minute warm up. Each participant then was asked to accelerate exercising to reach the specified minimum heart rate range corresponding to the exercise intensity within 5 minutes and required to keep the heart rate within the specified range for 15 minutes. The heart rate of each participant was recorded every three minutes. Participants were asked to continue to exercise for 5 minutes for cool down. During the recovery, the heart rate of each participant was recorded again.

#### 2.2.3. Posttest of Response Inhibition

Immediately after exercise intervention, the posttest of response inhibition was carried out for the individual. The posttest process was same as the pretest.

### 2.3. Study 2

**Figure 3:**
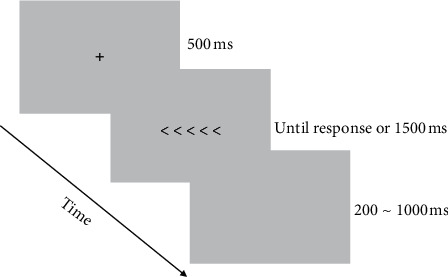
Process of each trial in study 2.

Study 2 included 15 college students with smartphone addiction. There were 8 males and 7 females with an average age of 19.87 ± 0.99 years. In pretest, the Flanker task [26] was used to measure the interference control of the participants. The independent variable was acute aerobic exercise intensity of three levels: high, moderate, and low intensity. The dependent variables were RT and accuracy. The process of each trial is shown in Figure 3. At the beginning of each trial, “+” was presented on the center of the screen, and participants were required to gaze for 500 ms. Then, a group of horizontally arranged stimulus symbols were randomly presented. The central stimulus was set as the target, and other stimuli on both sides were set as distractions. The criterion of meeting consistency between the orientation of the target and the orientation of distractions of the target was used to distinguish consistent trials from inconsistent trials. In consistent trials, >>>>> or <<<<< were presented, while in inconsistent trials, >><>> or <<><< were presented. Participants were then required to judge the orientation of the target as soon as possible under the premise of accuracy. If the target was <, the “*F*” key was pressed, but if the target was >, the “*J*” key was pressed. Until response or 1500 ms, one trial was completed, and the next trial was started after the interval of 200～1000 ms. Each participant had opportunities to practice, but the formal experiment could be allowed to carry out only when the accuracy was over 90%. In the formal test, 120 trials were carried out with 30 trials in each group of stimulus symbols. The exercise intervention for study 2 was the same as study 1. Immediately after exercise intervention, the posttest of response inhibition was carried out for the individual. The posttest process was the same as the pretest.

### 2.4. Statistical Analysis

Descriptive statistics were used to summarize measures of the RT and accuracy before and after acute aerobic exercise intervention in study 1 and 2. Those data with more than ±3SD were eliminated. The Kolmogorov–Smirnov test and Shapiro–Wilk test were performed to test the normality. The distributions were normal for the accuracy of NoGo stimulus (*p*.=0.967) and the RT of Go stimulus (*p*.=0.336) in study 1 and for the RT (*p*.=0.200) in study 2. Thus, analysis of variance (ANOVA) was conducted for further analyses. However, the normal distributions did not show for the accuracy of the Go stimulus (*p*.≤0.001) in study 1 and for the accuracy (*p*.≤0.001) in study 2. Therefore, we did normalizations first and then conducted ANOVA for further analyses.

To examine the effect of an acute aerobic exercise on response inhibition of college students with smartphone addiction, two-factor mixed design ANOVA, 3 (exercise intensity: high/moderate/low) × 2 (time: pretest/posttest), was conducted to assess variables of the accuracy of the NoGo stimulus and the RT of the Go stimulus and the accuracy of the Go stimulus. If there was a significant interaction between exercise intensity and time, simple effect analysis was applied to analyze whether there was a significant difference between pretest and posttest in three groups. If there was a significant difference in at least one group, one-way ANOVA was used to compare mean differences in three groups for evaluating the impact of different exercise intensities on response inhibition of college students with smartphone addiction.

For the effect of an acute aerobic exercise on interference control of college students with smartphone addiction, an ANOVA same as the above, 3 (exercise intensity: high/moderate/low) × 2 (time: pretest/posttest) × 2 (orientation: consistent/inconsistent), was conducted for the RT and was conducted for the accuracy. Three specific interaction contrasts were compared to test whether differences between consistent trials and inconsistent trials existed in pretest, posttest, and three groups.

Statistical analyses were performed using SPSS version 20 software for Windows. All data are reported as mean ± standard deviation (SD). A *p*. value less than 0.05 was considered to be statistically significant.

## 3. Results

### 3.1. Study 1

Figure 1 demonstrates the consort diagram of the study. The participants had a mean age of 20.20 ± 0.94 years. The results for the accuracy of NoGo stimulus response to pre- and postacute aerobic exercise are shown in Table 1. A two-way ANOVA (3 (exercise intensity: high/moderate/low) × 2 (time: pretest/posttest)) was conducted to evaluate the effect of an acute aerobic exercise and time on the response inhibition. The independent variable was the exercise intensity, which was divided into three levels: high, moderate, and low intensity. The dependent variables were reaction time (RT) and accuracy. The results of ANOVA indicated a significant time main effect, *F* = 63.38, *p*.≤0.001, *η*_*p*_^2^ = 0.84, as well as a significant exercising-by-time interaction effect, *F* = 4.03, *p*.=0.046, *η*_*p*_^2^ = 0.40. The main effect that was associated with the exercise intensity, however, was nonsignificant, *F* = 3.16, *p*.=0.079, *η*_*p*_^2^ = 0.35.

As shown in Table 1, the results showed that the mean accuracy of the NoGo stimulus was significantly different between pre- and posttest in all three intensity groups of study 1. These results suggested that, after an acute aerobic exercise, the accuracy of the NoGo stimulus could increase for these participants with smartphone addiction. To compare the effects of three exercise intensities on the accuracy of the NoGo stimulus, a one-way ANOVA was conducted to assess differences between pretest and posttest in three groups. The results were significant, *F* = 20.24, *p*=0.012. There were significant differences in the means between the S1LI group and the S1MI group (*p* ≤ 0.001) and between the S1MI and S1HI groups (*p*=0.001), but no significant differences between the S1LI and S1HI groups (*p*=0.075). The group that applied a moderate intensity showed the greatest increase in the accuracy of the NoGo stimulus in comparison to the other two groups. These results suggested that different levels of an acute aerobic exercise had a different effect on the accuracy of the NoGo stimulus in individuals, with the moderate intensity eliciting the largest effect.

Table 1 also presents the results for the RT and accuracy of Go stimulus response to pre- and postacute aerobic exercise. For the RT, the ANOVA analytic results (3 (exercise intensity: high/moderate/low) × 2 (time: pretest/posttest)) showed a significant time main effect, *F* = 439.27, *p*.≤0.001, *η*_*p*_^2^ = 0.97, as well as a significant interaction effect between time and exercise intensity, *F* = 89.99, *p*.≤0.001, *η*_*p*_^2^ = 0.94. However, the exercise intensity main effect was nonsignificant for the RT of the Go stimulus, *F* = 0.56, *p*.=0.588, *η*_*p*_^2^ = 0.09.

The results in Table 1 again showed that the mean RT of the Go stimulus decreased significantly in all three intensity groups as responding to the acute aerobic exercise intervention. These results implied that the participants could improve the RT of the Go stimulus after an acute aerobic exercise. A one-way ANOVA was conducted to evaluate effects of three exercise intensities on the mean RT of the Go stimulus between pretest and posttest. The results showed the significant differences between S1LI and S1MI groups (*p*.≤0.001), S1LI and S1HI groups (*p*.=0.001), and S1MI and S1HI groups (*p*.≤0.001), with the biggest change in the S1MI group, the second in the S1HI group, and the least in the S1LI group. These results indicated that the RT of the Go stimulus decreased significantly after an acute aerobic exercise and the moderate intensity was most effective than the low and high intensities.

For the accuracy of the Go stimulus, the ANOVA results (3 (exercise intensity: high/moderate/low) × 2 (time: pretest/posttest)] showed a significant time main effect, *F* = 10.81, *p*. = 0.006, *η*_*p*_^2^ = 0.47, suggesting that the accuracy of the Go stimulus increased in all three groups after an acute aerobic exercise. However, they were nonsignificant for the exercise intensity main effect and interaction effect between time and exercise intensity.

### 3.2. Study 2

The participants in study 2 had an average age of 19.87 ± 0.99 years.  Table 2 shows the RT and accuracy of consistent trial and inconsistent trial response to the three different exercise intensities before and after an acute aerobic exercise. A three-way mixed-design analysis of variance (3 (exercise intensity: high/moderate/low) × 2 (time: pretest/posttest) × 2 (orientation: consistent/inconsistent)) was conducted to evaluate the effect of an acute aerobic exercise and time on the response inhibition. The ANOVA results for the RT indicated a significant time main effect, *F* = 92.25, *p*.≤0.001, *η*_*p*_^2^ = 0.89, as well as a significant orientation effect, *F* = 29.31, *p*.≤0.001, *η*_*p*_^2^ = 0.71. However, all tests associated with the exercise intensity and interaction effects were nonsignificant. These results indicated that, after acute aerobic exercise, the RT of consistent and inconsistent trials decreased in all three groups and the independence of the acute aerobic exercise and the RT of inconsistent trials was significantly longer than that of consistent trials. For the accuracy, the results of ANOVA showed not significance for all main effects and the interaction effects, showing that the accuracy of consistent and inconsistent trials did not change before and after acute aerobic exercise in all three groups.

## 4. Discussion

### 4.1. Study 1

In study 1, we applied the Go/NoGo task to explore the effect of an acute aerobic cycling exercise at three intensity levels on response inhibition in college students with smartphone addiction. The Go/NoGo task is a common paradigm to measure response inhibition; however, the accuracy of the NoGo stimulus is usually considered as an indicator of response inhibition. The results showed that the accuracy of the NoGo stimulus was significantly increased after the exercise at high, moderate, and low intensity, indicating that 30 minutes of acute aerobic exercise could improve response inhibition for these smartphone-addicted individuals. Our results showed that compared with high and low intensity, moderate intensity elicited more significant effects on response inhibition. This finding was consistent with the previous studies on children and adults [19–23], suggesting that the inverse-U-curve relationship between the acute aerobic exercise intensity and inhibitory control also existed in college students with smartphone addiction.

As Diamond pointed, response inhibition means top-down self-control, involving in controlling over one's behaviour, staying on task despite distractions, or completing a task despite temptations to move on to a more interesting work [18]. According to the Strength Model of Self-Control, the exertion of self-control appears to depend on a limited energy resource [35]. The arousal mechanism in the reticular activation system could be activated by exercise [36, 37]. Particularly with the stress of a moderate-intensity exercise, the individuals were at the best level of arousal [28]. In this way, they could flexibly use top-down inhibitory control to overcome the dominant response to the NoGo stimulus [38].

With regard to responses to the RT of the Go stimulus, this study found that it was significantly reduced after the exercise at all three intensity levels. In addition, the most obvious reduction was also observed in the moderate-intensity group, demonstrating again that, with the moderate-intensity exercise, individuals would maintain at the best level of arousal and the task performance could be promoted to the greatest extent. However, while the accuracy of the Go stimulus was very high in all three groups, changes before and after acute aerobic exercises were not significant. It was possibly explained by the fact that the Go stimulus could account for 75%. As a result, it was very easy to respond based on individual habit and to reach a ceiling effect.

### 4.2. Study 2

By using the Flanker task, study 2 explored the effect of acute aerobic exercise on interference control for the college students with smartphone addiction. The Flanker task is a common paradigm to measure cognitive conflicting. The different RT and/or accuracy between consistent trials and inconsistent trials is usually considered as an indicator of conflicting effect. That is, if there was a conflicting effect, the different RT and/or accuracy between consistent trials and inconsistent trials would be significant. Similarly, if an acute aerobic exercise had an effect on interference control, significant different changes in the conflicting effect would be observed between pre- and postacute aerobic exercise for these tested individuals. The results of RT showed that there was a significant conflicting effect before and after acute aerobic exercise, but there was no significant difference between the two conflicting effects before and after exercise, implying that acute aerobic exercise had no influence on the change of interference control.

According to Diamond, selective attention and cognitive inhibition are two aspects of interference control. Empirically, the two could hardly be more tightly linked with the working memory (WM) [18]. Selective attention is interference control at the level of perception, which enables us to focus on what we choose and suppress attention to other stimuli. Keeping attention that focused on some mental contents might as easily be called focusing on information to be held in WM. Cognitive inhibition is considered to do suppressing proponent mental representations, keeping irrelevant information out, deleting no-longer-relevant information from the WM, and protecting the WM's mental workspace. As such, interference control and the WM would generally need one another and co-occur [18].

Müller [39] pointed out that the impairment of memory ability and the formation of related situational memory may be the important causes of behavioral addiction. Some studies on Internet addiction showed that Internet addicts had defects of working memory [40, 41]. Unfortunately, smartphone addiction has been also considered to have similar symptoms and behavioral defects as Internet addiction [14, 42]. Hadlington [42] reported that individuals with excessive mobile phone usage had the exposure of the electromagnetic radiation for a long time, which may weaken their cognitive functions such as working memory capacity and attention control. Earlier studies found that acute aerobic exercise could promote working memory [19, 43–46]. Most of these studies, however, were based on healthy human participants. Individuals with higher level of problematic mobile phone use have reduced cognitive resources; thus, competition arises between the need to conduct activities and the draw of excessive mobile phone use, which leads to commit blunders, memory errors, and components of distraction [42]. The results in this context showed no effect on interference control from acute aerobic exercise for the smartphone-addicted college students. One possible explanation is that they may have a lower WM capacity so as to lead to poorer AC and limit their capacity to prevent distraction [42].

Another closely related concept is mind wandering, in which attention switches from a current task to unrelated thoughts and feelings [47]. Early studies indicated that the relationship between mind wandering and attention executive control was bidirectional [48]. However, when the individual's attention executive control function detects their own thinking state, it can inhibit the generation of mind wandering. But on the one hand, mind wandering as a kind of interference will also affect the function of attention executive control. According to the Context Regulation Hypothesis, effective executive control can suppress self-generated thought when demands of external tasks are high. However, when external demands are low, executive control will take advantage of the person's excess resources and indulge in mind wandering [47]. A recent study found that task performance of smartphone addicts was more susceptible to high-frequency mind wandering [49]. The key to realize the generation of mind wandering and start executive control to restraining mind wandering lies in metacognition [47]. It is reported that both alcohol and cigarette are likely to cause more mind wandering without metacognition, induce poor task performance, and decrease in metacognition that is due to the execution failure caused by the desire for addicts [50, 51]. Considering the relationship between interference control and mind wandering, it is possible that smartphone addiction may damage metacognition, which is the same as alcohol and cigarette addiction, making the improvement of interference control be more difficult than that of response inhibition. As such, it is possibly an explanation why the acute aerobic exercise in this study promoted response inhibition but had no effect on interference control in college students with smartphone addiction. Nevertheless, the real mind wandering and metacognition of smartphone addicts responding to exercise need to be further investigated.

### 4.3. Limitations

The subjects in this study used power bicycle to do an acute aerobic exercise as intervention. Previous studies have shown that different exercise modalities may have different effects on inhibition function. Future research should consider using a treadmill and/or other types of exercise interventions to investigate the effect of acute aerobic exercise on the inhibition function in college students with mobile phone addiction. It can explore more aspects regarding responses of psychological functions to different exercises. This would also provide more choices for individuals with mobile phone addiction to motivate them participate in exercise and sports. In addition, influenced by COVID-19, this study failed to recruit enough subjects, leading to a small sample size for each group. Although the study produced good effect size and other indicators statistically, the future research needs to have a large sample size to elicit more powerful results and evidence.

## 5. Conclusions

In this study, we found that college students with smartphone addiction could effectively promote their response inhibition function by conducting an acute aerobic cycling exercise. This exercise at moderate intensity, compared to low or high intensity, had a most significant effect on the response inhibition. While the acute aerobic exercise appeared to have no effect on interference control, further research is needed. Our results suggest that the acute aerobic exercise is beneficial in promoting the inhibitory control for college students with smartphone addiction.

## Figures and Tables

**Table 1:** Data for the accuracy of the NoGo stimulus and the RT and accuracy of the Go stimulus.

Variables	Time	Low intensity	Moderate intensity	High intensity
Accuracy of the NoGo stimulus (%)	Pretest	74.33 ± 2.98	76.33 ± 8.03	71.67 ± 5.18
	Posttest	83.00 ± 2.72	96.33 ± 6.38	83.33 ± 3.20
	Difference	8.67 ± 6.50	20.00 ± 7.13	11.66 ± 7.90
	*F*	8.78	46.75	15.91
	*p*.	0.012	≤0.001	0.002

RT of the Go stimulus (ms)	Pretest	319.92 ± 51.59	348.11 ± 40.25	306.41 ± 20.89
	Posttest	307.25 ± 57.51	271.66 ± 34.83	271.60 ± 18.39
	Difference	12.67 ± 8.99	76.45 ± 6.77	34.81 ± 6.93
	*F*	13.76	501.47	104.01
	*p*.	0.003	≤0.001	≤0.001

Accuracy of the Go stimulus (%)	Pretest	99.22 ± 0.63	98.22 ± 1.90	98.33 ± 1.28
	Posttest	99.78 ± 0.31	100.00 ± 0.00	99.44 ± 0.78

*Note.* Values are means ± SD (*n* = 15). F and *p*. values were determined by simple effect analysis. A *p* value ≤0.05 was considered to be statistically significant.

**Table 2:** Data for the RT and accuracy of consistent trials and inconsistent trials.

		RT (ms)		Accuracy (%)	
Groups	Trials	Pretest	Posttest	Pretest	Posttest
Low intensity	Consistent	460.08 ± 10.13	424.88 ± 40.68	100.00 ± 0.00	100.00 ± 0.00
	Inconsistent	505.39 ± 24.86	484.04 ± 34.01	98.33 ± 3.33	100.00 ± 0.00

Moderate intensity	Consistent	453.42 ± 25.57	422.42 ± 27.15	100.00 ± 0.00	100.00 ± 0.00
	Inconsistent	492.36 ± 23.29	469.66 ± 34.47	99.17 ± 1.67	100.00 ± 0.00

High intensity	Consistent	458.09 ± 24.09	419.62 ± 45.44	100.00 ± 0.00	100.00 ± 0.00
	Inconsistent	506.48 ± 17.63	466.08 ± 44.46	99.17 ± 1.67	100.00 ± 0.00

*Note.* Values are means ± SD (*n* = 15). RT = reaction time. There were no significant differences (*p* ≤ 0.05) between different conditions in study 2.

## Data Availability

All data generated or analysed during this study are included in this published article.
